# Genome-Wide Meta-Analysis Identifies Two Novel Risk Loci for Epilepsy

**DOI:** 10.3389/fnins.2021.722592

**Published:** 2021-08-12

**Authors:** Meng Song, Jiewei Liu, Yongfeng Yang, Luxian Lv, Wenqiang Li, Xiong-Jian Luo

**Affiliations:** ^1^Henan Mental Hospital, The Second Affiliated Hospital of Xinxiang Medical University, Xinxiang, China; ^2^Henan Key Lab of Biological Psychiatry, International Joint Research Laboratory for Psychiatry and Neuroscience of Henan, Xinxiang Medical University, Xinxiang, China; ^3^Key Laboratory of Animal Models and Human Disease Mechanisms of the Chinese Academy of Sciences and Yunnan Province, Kunming Institute of Zoology, Chinese Academy of Sciences, Kunming, China; ^4^Center for Excellence in Animal Evolution and Genetics, Chinese Academy of Sciences, Kunming, China; ^5^KIZ-CUHK Joint Laboratory of Bioresources and Molecular Research in Common Diseases, Kunming Institute of Zoology, Chinese Academy of Sciences, Kunming, China

**Keywords:** epilepsy, GWAS, meta-analysis, TWAS, *GRM3*

## Abstract

Epilepsy (affects about 70 million people worldwide) is one of the most prevalent brain disorders and imposes a huge economic burden on society. Epilepsy has a strong genetic component. In this study, we perform the largest genome-wide meta-analysis of epilepsy (*N* = 8,00,869 subjects) by integrating four large-scale genome-wide association studies (GWASs) of epilepsy. We identified three genome-wide significant (GWS) (*p* < 5 × 10^–8^) risk loci for epilepsy. The risk loci on 7q21.11 [lead single nucleotide polymorphism (SNP) rs11978015, *p* = 9.26 × 10^–9^] and 8p23.1 (lead SNP rs28634186, *p* = 4.39 × 10^–8^) are newly identified in the present study. Of note, rs11978015 resides in upstream of *GRM3*, which encodes glutamate metabotropic receptor 3. *GRM3* has pivotal roles in neurotransmission and is involved in most aspects of normal brain function. In addition, we also identified three genes (*TTC21B*, *RP11-375N15.2*, and *TNKS*) whose *cis-*regulated expression level are associated with epilepsy, indicating that risk variants may confer epilepsy risk through regulating the expression of these genes. Our study not only provides new insights into genetic architecture of epilepsy but also prioritizes potential molecular targets (including *GRM3* and *TTC21B*) for development of new drugs and therapeutics for epilepsy.

## Introduction

Epilepsy is a common neurological disease characterized with recurrent unprovoked seizures. As one of the most prevalent brain disorders, epilepsy imposes a huge economic burden on society and affects about 70 million people worldwide (Thijs et al., 2019). Accumulating evidence indicate that epilepsy has a strong genetic component (Kjeldsen et al., 2003; Speed et al., 2014; Koeleman, 2018). Twin studies showed that genetic factors (total heritability) account for about 80% of the liability to epilepsy (Famula et al., 1997; Kjeldsen et al., 2003). Recent genome-wide association study (GWAS) estimated that the single nucleotide polymorphism (SNP) heritability (the proportion of variance in liability that can be attributed to SNPs, *h*^2^_*SNP*_) of genetic generalized epilepsy and focal epilepsy is about 32.1 and 9.2%, respectively (The International League Against Epilepsy Consortium on Complex Epilepsies, 2018), further indicating the pivotal role of common genetic variation in epilepsy. A recent study by Speed et al. (2014) also estimated that common variants collectively explain about 26% of phenotypic variation for all epilepsy. Despite the high heritability of epilepsy, only limited risk variants and loci have been identified by large-scale genetic studies to date (International League Against Epilepsy Consortium on Complex Epilepsies, 2014; The International League Against Epilepsy Consortium on Complex Epilepsies, 2018).

To further identify risk variants and to uncover the missing heritability of epilepsy, in this study, we report the largest genome-wide meta-analysis of epilepsy (*N* = 8,00,869 subjects) by integrating four large-scale GWASs of epilepsy. The first GWAS (14,534 cases and 24,218 controls) was from a recent study by the International League Against Epilepsy Consortium on Complex Epilepsies (ILAE Consortium) (The International League Against Epilepsy Consortium on Complex Epilepsies, 2018). The second GWAS was from the UK Biobank (Sudlow et al., 2015), genome-wide summary statistics of epilepsy in UK Biobank [5,087 epilepsy cases (Phecode:X345) and 3,95,209 controls] generated by the scalable and accurate implementation of generalized mixed model (SAIGE) (Zhou et al., 2018) were used in this study. The third GWAS was from a recent study by Ishigaki et al. (2020) (2,143 epilepsy cases and 2,10,310 controls). The fourth GWAS dataset was from the FINNGEN^1^ [4,588 epilepsy cases (phenocode: G6_EPLEPSY) and 1,44,780 controls].

## Materials and Methods

### Epilepsy GWAS From the ILAE Consortium

International League Against Epilepsy Consortium (The International League Against Epilepsy Consortium on Complex Epilepsies, 2018) conducted a large-scale *trans-*ethnic meta-analysis (15,212 epilepsy cases and 29,677 controls) by combining genome-wide associations conducted in Caucasians, Asians, and Africans. Epilepsy cases were classified into three broad categories and seven subtypes. In this study, we used the genome-wide associations from all epilepsy cases (including focal epilepsy, genetic generalized epilepsy, and unclassified epilepsy). In addition, considering that the number of cases in Asian (*n* = 531) and African (*n* = 147) GWASs were quite small, only associations from Europeans (14,534 cases and 24,218 controls) were included in our study. Detailed information about diagnosis and classification of epilepsy cases, genotyping, imputation, and quality controls have been described in the original study (The International League Against Epilepsy Consortium on Complex Epilepsies, 2018). The linear mixed model implemented in BOLT-LMM (Loh et al., 2015) was used to test the associations between genetic variants and epilepsy. As the effect size reported by BOLT-LMM is a Beta coefficient, we transformed Beta values into odds ratio (OR) using the method developed by Lloyd-Jones et al. (2018).^2^ The parameters used for transformation are as follows: *k* (the prevalence of epilepsy in the GWAS sample) = 0.38 (14,534/38,752). Allele 1, allele 2, allele frequency, Beta, SE, and *p*-values were adopted from the original study (The International League Against Epilepsy Consortium on Complex Epilepsies, 2018). The SE of ln(OR) was calculated using the following formula: SE = ln(OR)/*Z* (where *Z*-value was calculated using qnorm function implemented in R software (v3.4.1).

### Epilepsy GWAS From UK Biobank

UK Biobank is an open access resource that aims to identify the associations between common genetic variation and multiple complex diseases and traits (Sudlow et al., 2015; Bycroft et al., 2018). Detailed information about UK Biobank have been described previously (Bycroft et al., 2018). In this study, we used the summary statistics of epilepsy GWAS from the PheWeb (Gagliano Taliun et al., 2020).^3^ Briefly, SAIGE (Zhou et al., 2018) was used to test the associations between genetic variants and phenotypes included in UK Biobank. The phenotype code for epilepsy in PheWeb is X345 (corresponding to ICD9 345, or ICD10 code G40). A total of 5,087 epilepsy and 3,95,209 controls were included in GWAS. Unlike the ILAE Consortium, epilepsy cases in UK Biobank were not divided into focal and generalized epilepsy. We transformed the Beta effect size into OR using the following formula: OR = exp^(Beta)^. In addition, SNPs with minor allele frequency (MAF) of less than 0.01 were excluded. More detailed information about the UK Biobank and PheWeb can be found in previous papers (Bycroft et al., 2018; Zhou et al., 2018; Gagliano Taliun et al., 2020).

### Epilepsy GWAS of Japanese Population

The genome-wide associations of Japanese population were from the study of Ishigaki et al. (2020). Briefly, Ishigaki et al. (2020) conducted a GWAS of 42 diseases in a large-scale Japanese population. Associations between genetic variants and diseases were assessed using SAIGE (Zhou et al., 2018). The GWAS of epilepsy included in the study of Ishigaki et al. (2020) contained 2,143 cases and 2,10,310 controls. Summary statistics were downloaded from the Japanese ENcyclopedia of GEnetic associations by Riken (JENGER).^4^ Detailed information about the subjects, diagnosis, genotyping, imputation, quality control, and statistical analysis have been described in the study of Ishigaki et al. (2020). We transformed the Beta effect size into OR using the following formula: OR = exp^(Beta)^. Besides, SNPs with MAF of less than 0.01 were excluded for meta-analysis.

### Epilepsy GWAS From FINNGEN

FINNGEN is a large-scale and open access resource which aims to improve human health through genetic research. Its ultimate goal is to identify new therapeutic targets and diagnostics for treating human diseases. It was launched in 2017, and it will collect genome information and digital healthcare data of about 5,00,000 Finnish people. By integrating genetic resources and digital healthcare data from multiple organizations, including Finnish universities, biobanks, hospitals, and so on, this project expects to achieve breakthroughs in disease diagnosis, prevention, and treatment. GWASs in FINNGEN were also performed using SAIGE (Zhou et al., 2018) and summary statistics from data freeze four results [4,588 epilepsy cases (phenocode: G6_EPLEPSY) and 1,44,780 controls] of FINNGEN were used in this study. Detailed information about the subjects, diagnosis, genotyping, imputation, quality control, and statistical analysis have been described in the homepage of FINNGEN: https://www.finngen.fi/en. We transformed the Beta effect size into OR using the following formula: OR = exp^(Beta)^. Besides, SNPs with MAF of less than 0.01 were excluded for meta-analysis. Of note, the ILAE GWAS performed analysis on three subphenotypes of epilepsy (focal, GGE, and all epilepsy) (International League Against Epilepsy Consortium on Complex Epilepsies, 2014). In addition, cases in FinnGen included focal epilepsy, generalized epilepsy, and epilepsy (broader sense). However, the cases in Japanese and UK Biobank GWASs were not divided into subphenotypes (Ishigaki et al., 2020). Therefore, we did not divide the epilepsy into subphenotypes (i.e., we used all epilepsy cases) in this study.

### Meta-Analysis

For the other three datasets (including UK Biobank, Japanese GWAS, and FINNGEN; effect size was calculated using SAIGE, which uses logistic repression to perform association test), the effect size of each SNP was converted into OR using the following formula: OR = exp^Beta^. SNPs with MAF of less than 0.01 were excluded. Meta-analysis was performed using PLINK (V1.90) (Purcell et al., 2007), with the use of fixed-effect model. The inverse variance-based analysis to be implemented in PLINK was used for meta-analysis.

### Tissue- and Cell-Type Enrichment Analysis

We used MAGMA (de Leeuw et al., 2015) [implemented in FUMA (Watanabe et al., 2017)] to perform tissue- and cell-type enrichment analysis. MAGMA is a powerful tool for gene and gene-set analysis, and it uses GWAS summary statistics as input. MAGMA first derives a gene-level *p*-value by using a multiple linear principal component regression model. The gene-level *p*-values were then used for further gene-set analysis. To test if the genetic associations from GWASs are enriched in specific tissues, MAGMA utilizes gene expression data from the GTEx (53 human tissues) for tissue enrichment analysis.

We also conducted single-cell enrichment analysis by MAGMA followed by the methods of a recent published paper about Parkinson’s disease (Bryois et al., 2020). The single-cell RNA-seq data was from mouse central nervous system which include 1,60,769 single cells in total (Zeisel et al., 2018); top 10% genes that ranked by gene expression specificity of each cell type was remained for the MAGMA gene-set enrichment analysis (de Leeuw et al., 2015). For further detailed information about single-cell data generation, data processing, and gene expression specificity calculation, please refer to the original paper (Zeisel et al., 2018; Bryois et al., 2020). Detailed information about MAGMA, FUMA, and tissue- and cell-type enrichment analyses can be found in the original papers (de Leeuw et al., 2015; Watanabe et al., 2017; Zeisel et al., 2018; Bryois et al., 2020) and the FUMA website.^5^

### Transcriptome-Wide Association Study

Transcriptome-wide association study (TWAS) aims to identify genes whose genetically regulated expression level are associated with complex human diseases or traits (Gusev et al., 2016). TWAS firstly uses an external expression reference panel (which contained gene expression data and genome-wide SNPs) to establish SNP-expression weights (i.e., SNP-gene expression correlations). These SNP-expression weights are then used to predict the expression level of genes in individuals included in GWAS. Finally, statistical inferences are made to test if the expression level of a gene is associated with diseases or traits. Considering that epilepsy is a brain disorder, in this study, we utilized the gene expression and genotype data from the PsychENCODE (Gandal et al., 2018) as the reference panel to construct the SNP-gene expression weights. In brief, PsychENCODE integrated gene expression (from human brain tissues, most of tissues are the prefrontal cortex) and genotype data of over 2,000 human subjects. Gene expression was quantified with RNA sequencing, and genotypes were determined using SNP arrays. Among the subjects included in PsychENCODE, expression data, and genotypes of 1,321 indivuduals (only adult individuals with matching gene expression and genotypes can be used for expression quantitative trait loci (eQTL) analysis) were used for eQTL analysis and construction of SNP-gene expression weights. We used FUSION (Gusev et al., 2016) pipeline to prepare the SNP-gene expression weights. We integrated the constructed SNP-gene expression weights and GWAS summary statistic from the meta-analysis to conduct TWAS, with the use of default parameters and settings. Results of TWAS were corrected by the *Bonferroni* correction approach. More detailed information about TWAS and FUSION can be found in the original paper (Lloyd-Jones et al., 2018) and FUSION website.^6^

### Interaction Analysis Between the Risk Genes and Drugs

To explore if the identified risk genes may be targeted as potential therapeutic targets, we explored the interaction between the identified epilepsy risk genes and drugs using the drug-gene interactions database (DGIdb^7^) (Freshour et al., 2020). Briefly, DGIdb collected interactions between genes and drugs from several well-characterized databases, including DrugBank, Drug Target Commons, PharmGKB, Chembl, etc. In addition, pathways and molecular functions of genes were also considered. These combined information were then used to predict the interactions between genes and drugs.

## Results

**FIGURE 1:**
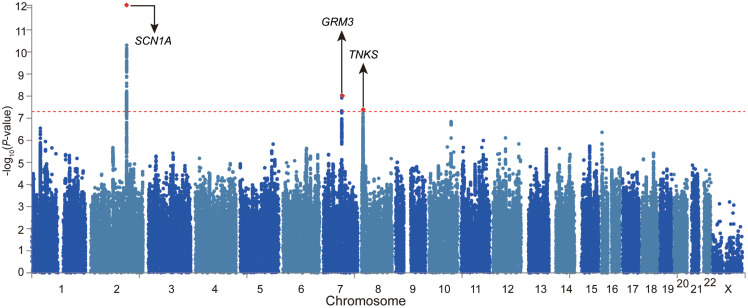
Meta-analysis of four large-scale genome-wide association studies (GWASs) identified two novel risk loci for epilepsy. Manhattan plot of the meta-analysis of epilepsy (26,352 cases and 7,74,517 controls). The risk loci on 7q21.11 (lead SNP rs11978015, *p* = 9.26 × 10^– 9^) and 8p23.1 (lead SNP rs28634186, *p* = 4.39 × 10^– 8^) are newly identified in the present study.

**FIGURE 2:**
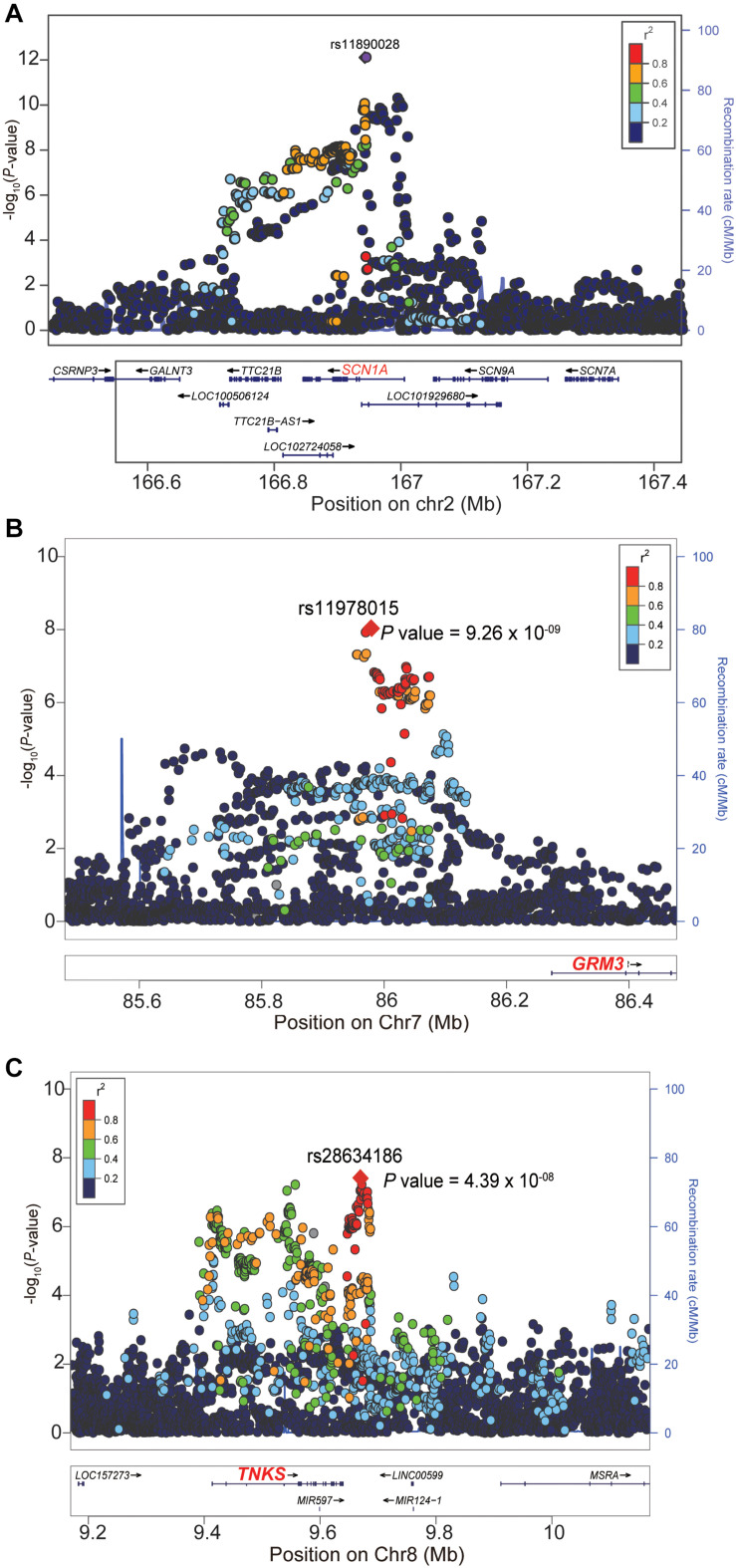
Locuszoom plots of the three genome-wide significant (GWS) loci. **(A)** The lead SNP rs11890028 (*P* = 7.76 × 10^– 13^) of the 2q24.3 risk locus resides in intronic region of *SCN1A*. **(B)** The lead SNP rs11978015 (*P* = 9.26 × 10^– 9^) of the 7q21.11 risk locus resides upstream of *GRM3*. **(C)** The nearest gene for the lead SNP rs28634186 (*P* = 4.39 × 10^– 8^) on the 8p23.1 is *TNKS*. The read dotted line represents the genome-wide significance level (5.0 × 10^– 8^).

We firstly examined the genome-wide significant (GWS) loci in each GWAS. For epilepsy GWAS from the ILAE Consortium, two loci reached GWS level (of note, this dataset contains three subtypes and we only included GWAS of all epilepsy) (The International League Against Epilepsy Consortium on Complex Epilepsies, 2018). For epilepsy GWAS from UK Biobank, Japanese population, and FINNGEN, no loci showed GWS associations with epilepsy (Supplementary Figures 1–3). We then conducted a genome-wide meta-analysis by combining the genome-wide association results of the four studies. The QQ plot is shown in Supplementary Figure 4. The lambda_GC_ (λ_1,000_, genomic control (GC) inflation lambda scaled for 1,000 cases and 1,000 controls) of our meta-analysis is 1.0027, indicating that the association signals were mainly driven by polynenicity rather than population structure. In this largest *trans-*ethnic meta-analysis (26,352 cases and 7,74,517 controls), we identified three GWS (*p* < 5 × 10^–8^) risk loci for epilepsy (Figure 1 and Table 1). The risk locus on 2q24.3 has been reported in a previous GWAS (The International League Against Epilepsy Consortium on Complex Epilepsies, 2018). Of note, the most significant variant (lead SNP rs11890028) on 2q24.3 resides in the intron 7 of *SCN1A*, a well-characterized risk gene for epilepsy (Claes et al., 2001; Parihar and Ganesh, 2013; International League Against Epilepsy Consortium on Complex Epilepsies, 2014; Figure 2B). However, the risk loci on 7q21.11 (lead SNP rs11978015, *p* = 9.26 × 10^–9^) and 8p23.1 (lead SNP rs28634186, *p* = 4.39 × 10^–8^) are newly identified in the present study (Figures 2B,C). Of note, rs11978015 resides in upstream of *GRM3* (Figure 2B), which encodes glutamate metabotropic receptor 3. *GRM3* has pivotal roles in neurotransmission and is involved in most aspects of normal brain function (Blacker et al., 2017; Chaki, 2017; Jin et al., 2018; Neale and Olszewski, 2019; Joffe et al., 2021; Kellner et al., 2021). Another GWS risk variant rs28634186 is located in intergenic region and the nearest gene for rs28634186 is *TNKS* (Figure 2C). Genetic correlation between epilepsy and other diseases [using LD Hub (Zheng et al., 2017)]^8^ showed significant correlations with amyotrophic lateral sclerosis (ALS), schizophrenia, and bipolar disorder (Supplementary Table 1).

**TABLE 1:** Genome-wide significant (GWS) loci identified in this study.

Locus	Lead SNP	Chr	Pos	A1/A2	*p*-Value	OR^*a*^	Nearby gene (s)
1	rs11890028	2	166,943,277	T/G	7.76e−13	1.085	*TTC21B, SCN1A*
2	rs11978015	7	85,977,972	G/A	9.26e−09	1.058	*GRM3*
3	rs28634186	8	9,669,335	T/C	4.39e−08	1.056	*TNKS*

*^*a*^OR is based on A1.*

**FIGURE 3:**
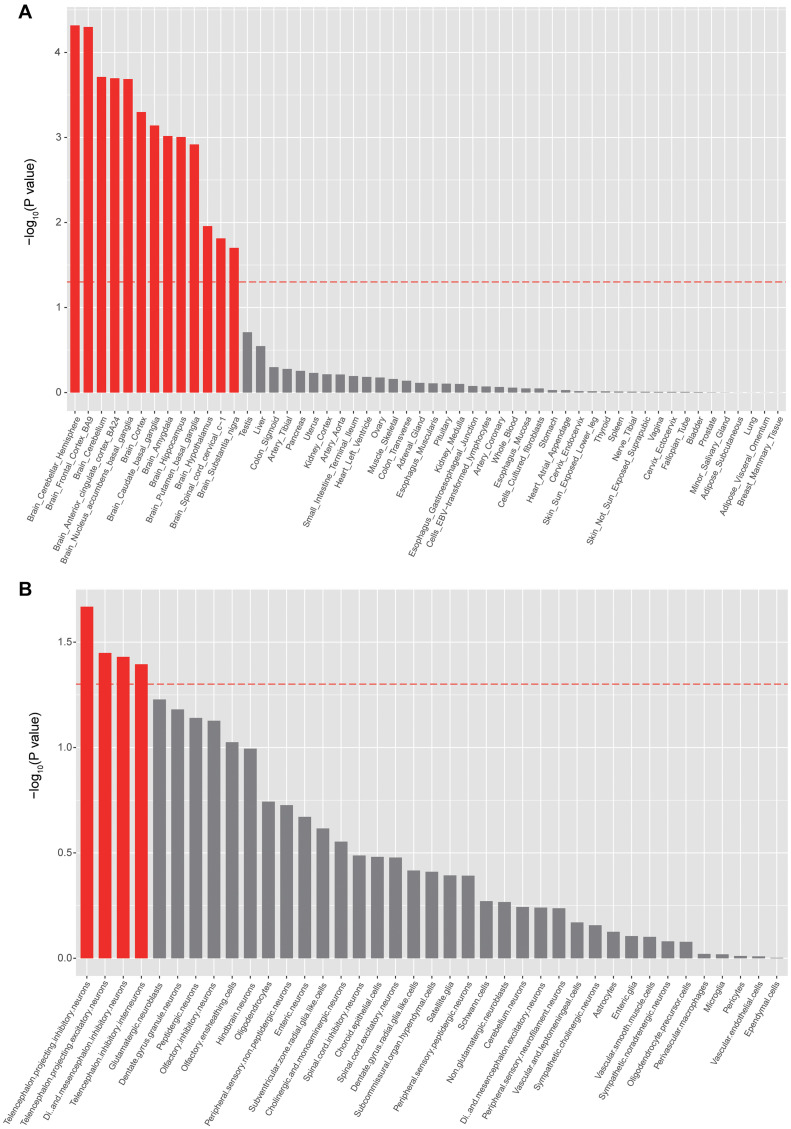
Tissue- and cell-type enrichments of epilepsy GWAS associations. **(A)** Tissues that showed significant enrichment (corrected *p* < 0.05) are shown in red. **(B)** Cell types that showed significant enrichment (corrected *p* < 0.05) are shown in red.

To identify the tissues and cell types that risk genes may exert their biological effects on epilepsy, we further performed tissue- and cell-type-specific enrichment analysis using MAGMA (see text footnote 5). As expected, the GWAS associations were significantly enriched in brain tissues (Figure 3A), with the highest enrichment in the cerebellar hemisphere and frontal cortex (Figure 3A). Cell-type-specific enrichment analysis showed significant enrichment of GWAS associations in telencephalon projecting inhibitory and excitatory neurons (Figure 3B).

**FIGURE 4:**
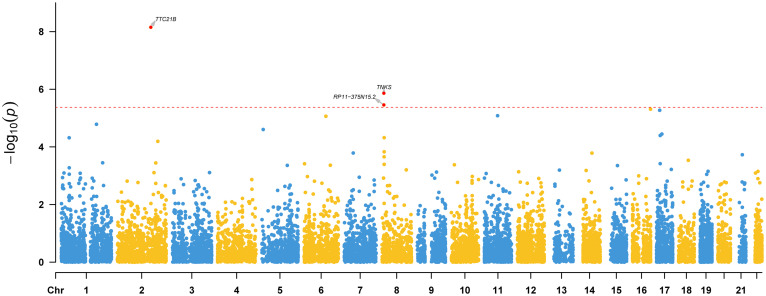
Transcriptome-wide association study (TWAS) results of epilepsy. Three genes (*TTC21B, RP11-375N15.2*, and *TNKS*) showed transcriptome-wide significant (TWS) associations with epilepsy, indicating that the genetically regulated expression of these genes are associated with epilepsy. Transcriptome-wide significance (corrected by Bonferroni adjustment) is marked by a red dotted line.

To identify risk genes whose genetically regulated expression change are associated with epilepsy, we further conducted a TWAS by integrating genome-wide summary statistics of epilepsy (from meta-analysis) and SNP-gene expression weights from the PsychENCODE (*N* = 1,371).^9^ Three genes (*TTC21B*, *RP11-375N15.2*, and *TNKS*) showed transcriptome-wide significant (TWS) associations (Bonferroni corrected *p* < 0.05) with epilepsy (Figure 4), indicating that risk variants may confer epilepsy risk through regulating the expression of these genes.

Finally, to explore if the risk identified may be targeted for epilepsy treatment, we further examined the interactions between the identified risk genes and drugs using DGIdb (Freshour et al., 2020). Our analysis showed that SCN1A interacts with many drugs (Supplementary Table 2), suggesting this gene may be targeted as a potential therapeutic target. In addition, GRM3 and TNKS also show interactions with several drugs (Supplementary Tables 3, 4). These data suggest that these three genes may be targeted for epilepsy treatment.

## Discussion

In summary, we performed the largest meta-analysis of epilepsy GWAS in this study and identified two new risk loci for epilepsy. We also showed that genetic associations of epilepsy are enriched in brain tissues and telencephalon projecting inhibitory and excitatory neurons. Of note, we identified three risk genes (*TTC21B*, *RP11-375N15.2*, and *TNKS*) whose expression perturbation may have a role in epilepsy. Interestingly, *TTC21B* resides in the 2q24.3 locus, a region that contains the well-characterized epilepsy risk gene *SCN1A* (Claes et al., 2001, 2009; Meng et al., 2013; Parihar and Ganesh, 2013; International League Against Epilepsy Consortium on Complex Epilepsies, 2014; Haigh et al., 2021). Although the genome-wide association signal (Figure 2A) and previous studies (Claes et al., 2001, 2009; Meng et al., 2013; Parihar and Ganesh, 2013; International League Against Epilepsy Consortium on Complex Epilepsies, 2014; Haigh et al., 2021) have clearly showed that *SCN1A* represents the most possible causal gene for this risk locus, our TWAS suggested that *TTC21B* may also have a potential role in epilepsy (Figure 4). In fact, previous studies also have revealed the potential role of *TTC21B* in epilepsy (Mirza et al., 2017; The International League Against Epilepsy Consortium on Complex Epilepsies, 2018). More work is needed to elucidate the role of *TTC21B* in epilepsy.

We noticed a second peak which is not in linkage disequilibrium (LD) with the main signal at 2q24.3 (located at the right of the top hit) (Figure 2A), suggesting two independent genetic signals at this locus. We thus performed a conditional analysis using genome-wide complex trait analysis (GCTA) (Yang et al., 2011). Conditional analysis on the top hit SNP rs11890028 suggested that the second peak might not be an independent signal. The *p*-value of rs11896706 (located at the right of the top hit, Figure 2A) conditioned on rs11890028 (the top hit) is 5.01 × 10^–5^. In addition, we also checked the LD pattern of genetic variants spanning this genomic region [using genotype data of Europeans from the 1,000 Genomes project (The 1000 Genomes Project Consortium, 2015)]. We found that the lead SNP (rs11890028) showed weak LD (*r*^2^ = 0.13) with SNP rs11896706 in this region, suggesting that the second signal peak in this locus was likely due to LD between the lead SNP rs11890028 and nearby variants. More work is needed to further investigate if two independent GWAS signals at this locus.

Intriguingly, our findings suggested that *GRM3* may be a potential risk gene for epilepsy. GRM3 encodes glutamate metabotropic receptor 3 (mGluR3), a member of the family of G protein-coupled receptors. As a major receptor of glutamate, postsynaptic GRM3 is crucial for mGluR3-dependent long-term depression (LTD) (Joffe et al., 2021) and cognitive function (Jin et al., 2018; Neale and Olszewski, 2019). The binding of glutamate to mGluR3 results in activation of G protein-coupled receptor, which in turn regulates gene transcription, release of neurotransmitter, neuron activity, and synaptic transmission (Blacker et al., 2017; Chaki, 2017; Kellner et al., 2021). In fact, a recent study has proposed that modulation of astrocyte glutamate uptake (and/or mGluR activation) may represent a potential therapeutic approach for epilepsy treatment (Peterson and Binder, 2020). These lines of evidence suggest that *GRM3* may have a role in epilepsy.

Our GWAS meta-analysis suggested that 8p23.1 is a risk locus for epilepsy. To further explore the potential risk gene in this locus, we searched literatures and performed additional analysis. Based on the results of GWAS and TWAS, we speculated that *TNKS* (also known as *PARP5A*) may be the potential risk gene at this locus. First, most of the significant variants identified in our GWAS meta-analysis are located in genomic region (including gene body and upstream of *TNKS*) containing *TNKS* (Figure 2C). Second, our TWAS results suggested that *TNKS* is a gene whose genetically regulated expression may have a role in epilepsy (Figure 4). Third, expression analysis using single-cell RNA-seq data showed that *TNKS* is highly expressed in different cell types of the brain (Supplementary Figures 5, 6). Fourth, the biological function also supports *TNKS* as a potential risk gene. *TNKS* encodes tankyrases [members of the poly(ADP-ribose) polymerase (PARP) family] that are involved in the regulation of Wnt/beta-catenin signaling (Bao et al., 2012; Kulak et al., 2015; Yang et al., 2019) and PTEN (Li et al., 2015). Considering the important role of Wnt/beta-catenin signaling (Bengoa-Vergniory and Kypta, 2015; Arnes and Casas Tinto, 2017; Noelanders and Vleminckx, 2017; Bem et al., 2019) and PTEN (Endersby and Baker, 2008; Zhou and Parada, 2012; Spina Nagy et al., 2021) in the brain, it is possible that *TNKS* confers risk of epilepsy by regulating Wnt/beta-catenin signaling pathway. In fact, recent studies also have showed that TNKS modulates TDP-43, a protein with a central role in ALS and frontotemporal degeneration (FTD) (McGurk et al., 2020; Tanji et al., 2021), further suggesting the pivotal role of *TNKS* in the human brain. Finally, Wnt/β-catenin signaling was proposed to be a potential target for epilepsy therapy (Huang et al., 2015; Hodges and Lugo, 2018). Collectively, these evidence suggest that *TNKS* may represent the potential risk gene at 8p23.1. However, further genetic studies and functional characterization are needed to validate if *TNKS* is a risk gene for epilepsy.

It should be noted that biobank data as a resource for epilepsy GWAS has weakness and limitations. Compared with traditional GWAS (GWASs usually use well-phenotyped cohort), the subjects included in biobank data are usually based on electronic records or questionnaire, which may affect the accurate phenotyping of the included individuals. In addition, the number of cases included in biobank data is usually much smaller than controls (case-control imbalance), which influences the statistic power of biobank data. Finally, population relatedness or structure also needs to be carefully considered and controlled in biobank data.

## Conclusion

In conclusion, our study not only provides new insights into genetic architecture of epilepsy but also prioritizes potential molecular targets (including *GRM3* and *TNKS*) for development of new drugs and therapeutics for epilepsy.

## Data Availability Statement

The original contributions presented in the study are included in the article/Supplementary Material, further inquiries can be directed to the corresponding author/s. Custom codes used (including Perl, R, and Unix) were used for data processing. These custom codes can be made available from the corresponding author upon request.

## Ethics Statement

The studies involving human participants were reviewed and approved by the ILAE Consortium, UK Biobank, JENGER, and FINNGEN. The patients/participants provided their written informed consent to participate in this study.

## Author Contributions

X-JL conceived, designed, and supervised the whole study and wrote and revised the manuscript. MS, X-JL, YY, LL, and WL collected the GWAS summary statistics, performed the data processing and transformation, and conducted the GWAS meta-analysis. JL performed the tissue and cell type-specific enrichments analyses, TWAS, and generated the Manhattan and locus zoom plots. LL provided critical comments for the manuscript improvement. All authors read this manuscript carefully, provided critical comments, and approved the manuscript.

## Conflict of Interest

The authors declare that the research was conducted in the absence of any commercial or financial relationships that could be construed as a potential conflict of interest.

## Publisher’s Note

All claims expressed in this article are solely those of the authors and do not necessarily represent those of their affiliated organizations, or those of the publisher, the editors and the reviewers. Any product that may be evaluated in this article, or claim that may be made by its manufacturer, is not guaranteed or endorsed by the publisher.

## Footnotes

1
https://www.finngen.fi/en

2
https://github.com/lukelloydjones/ORShiny

3
http://pheweb.sph.umich.edu/SAIGE-UKB/

4
http://jenger.riken.jp/en/

5
https://fuma.ctglab.nl/

6
http://gusevlab.org/projects/fusion/

7
https://www.dgidb.org/

8
http://www.nealelab.is/tools-and-software

9
http://www.psychencode.org/
