# Supplementary material for: Genome-Wide Meta-Analysis Identifies Two Novel Risk Loci for Epilepsy
Source: Front Neurosci. 2021 Aug 12;15:722592. doi: 10.3389/fnins.2021.722592 (PMC8397525; doi:10.3389/fnins.2021.722592)
Supplement: Supplementary file 1 [file Data_Sheet_1.docx]

**Supplementary material for**

**Genome-wide meta-analysis identifies two novel risk loci for epilepsy**

Meng Song, MD^1,3,6^; Jiewei Liu, PhD^2,6^; Yongfeng Yang, MD^1,3^; Luxian Lv, MD^1,3^; Wenqiang Li, MD^1,3,*^; Xiong-Jian Luo, PhD^2,4,5,*^

*To whom correspondence should be addressed: Xiong-Jian Luo, Key Laboratory of Animal Models and Human Disease Mechanisms, Kunming Institute of Zoology, Chinese Academy of Sciences, Kunming, Yunnan 650204, China; Tel: +86-871-68125413, Fax: +86-871-68125413, E-mail: luoxiongjian@mail.kiz.ac.cn (XJL).


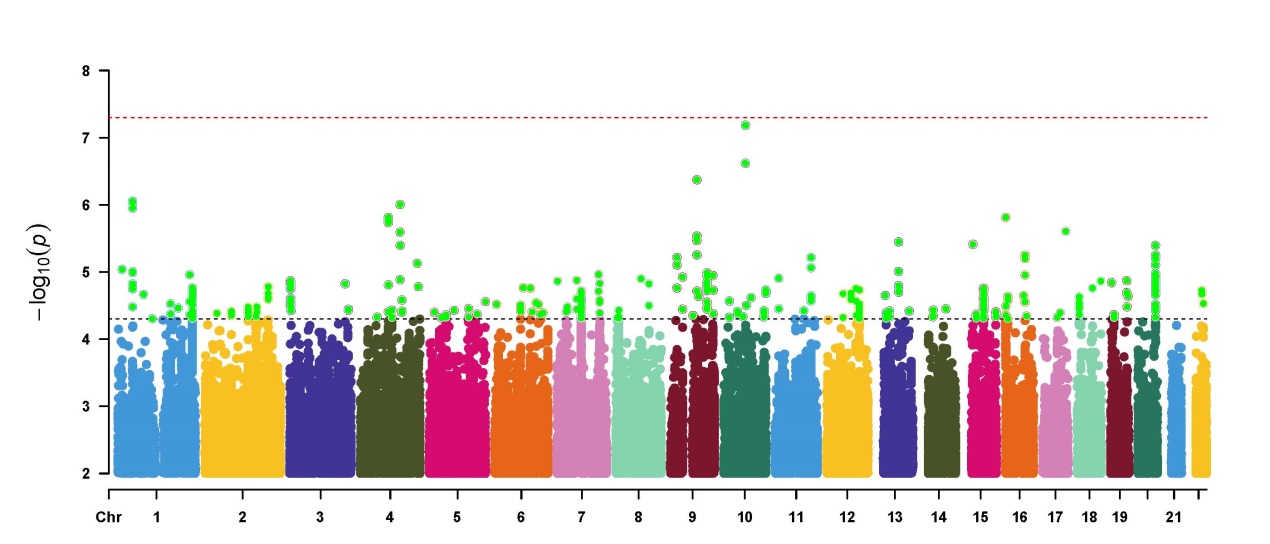


**Fig. S1. Manhattan plot of the epilepsy GWAS (UK Biobank).**


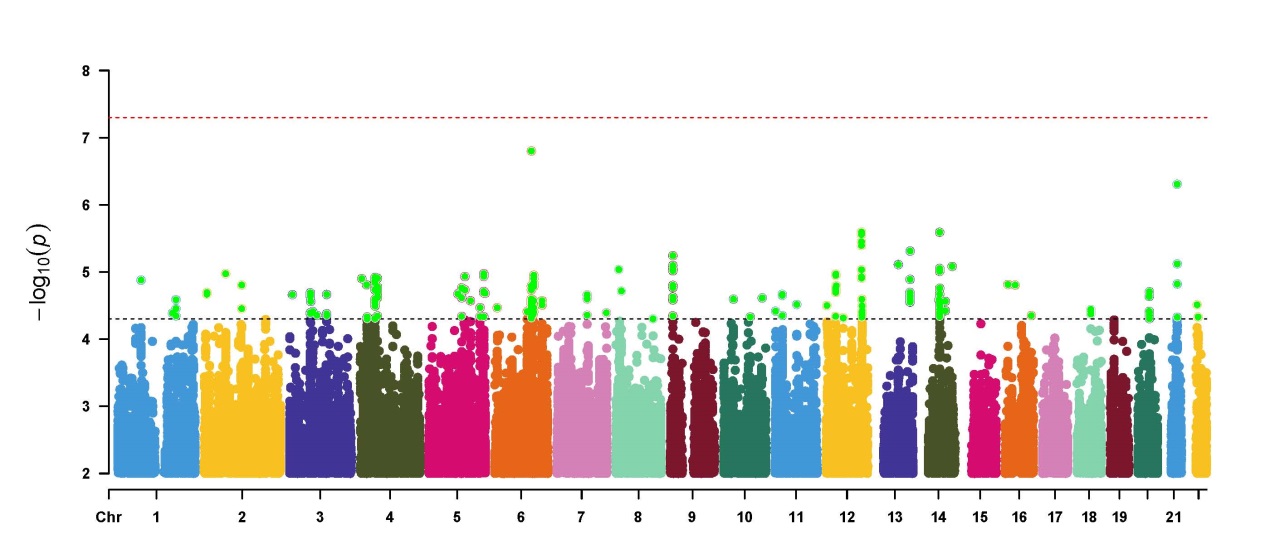
 **Fig.S2. Manhattan plot of the epilepsy GWAS (Japanese population).**


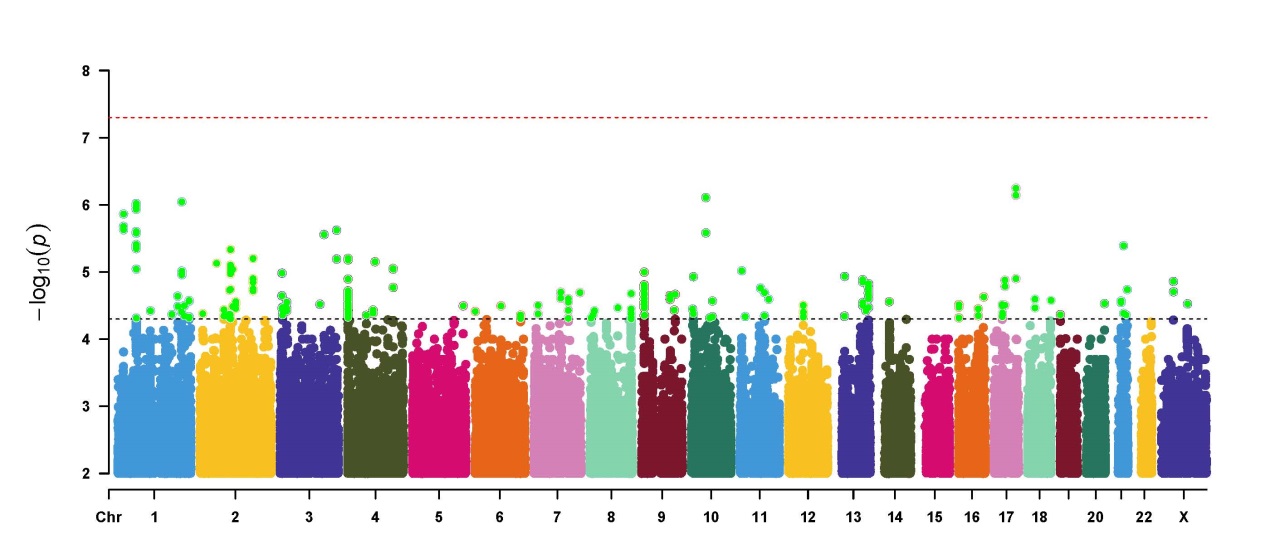


**Fig. S3. Manhattan plot of the epilepsy GWAS (FINNGEN).**


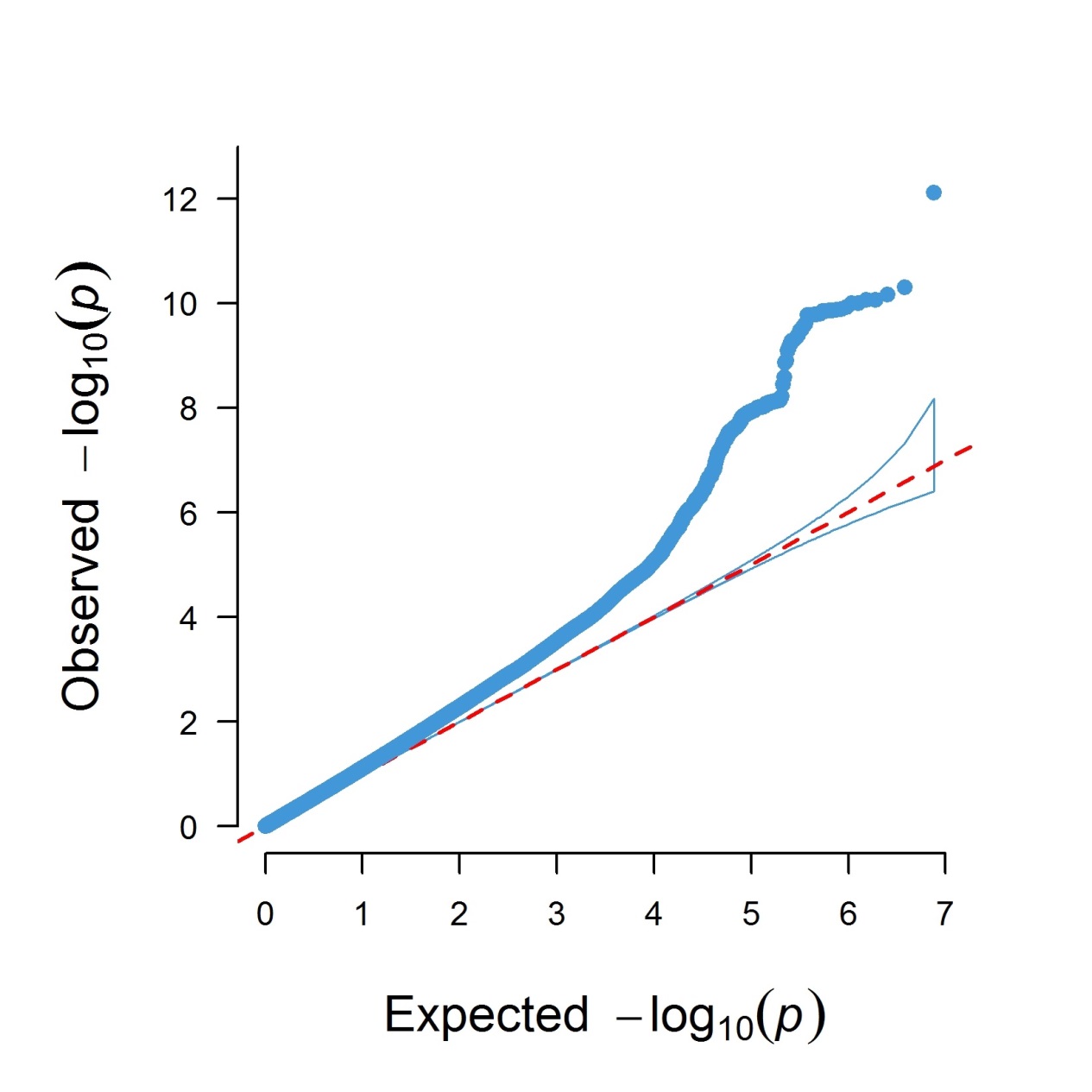


**Fig. S4. The QQ plot of the meta-analysis.**


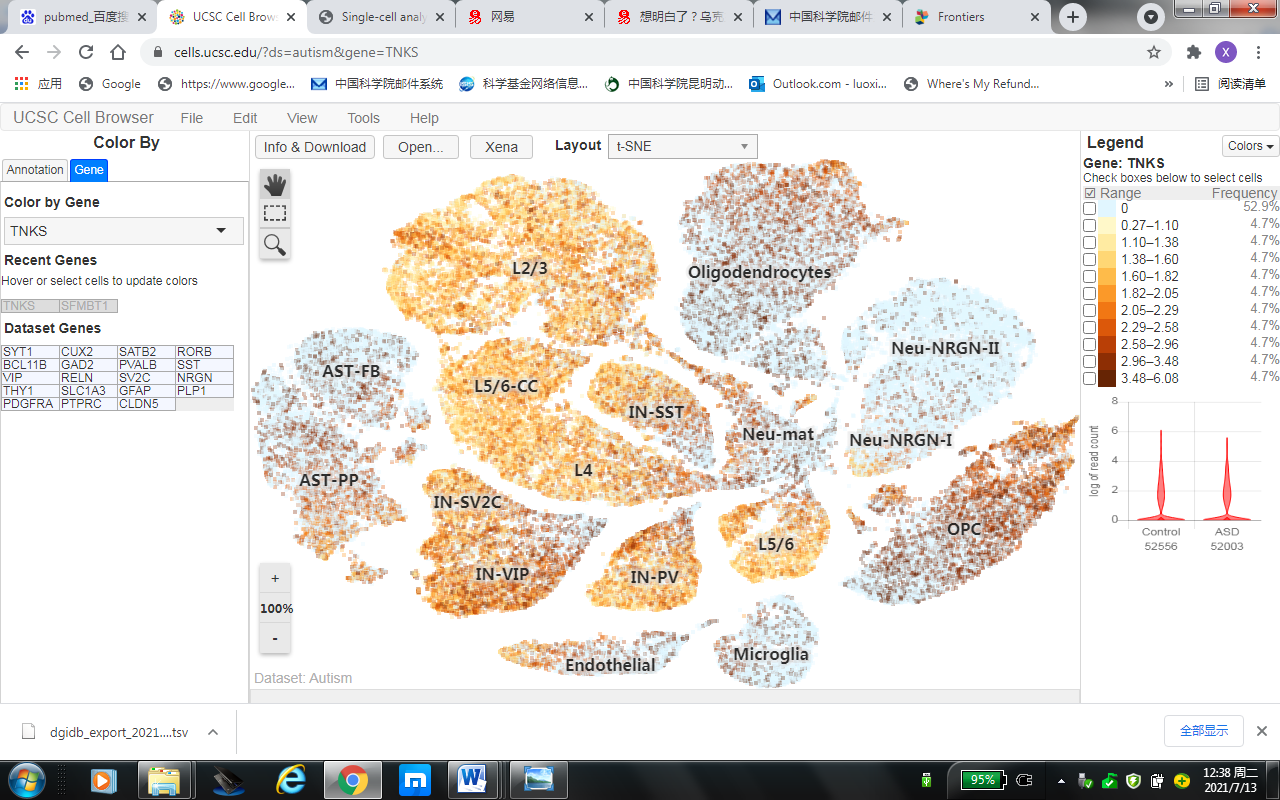


**Fig. S5. *TNKS* expression in different brain cell types.** Data was from the UCSC Cell Browser ([https://cells.ucsc.edu/?ds=autism&gene=TNKS#](https://cells.ucsc.edu/?ds=autism&gene=TNKS)).


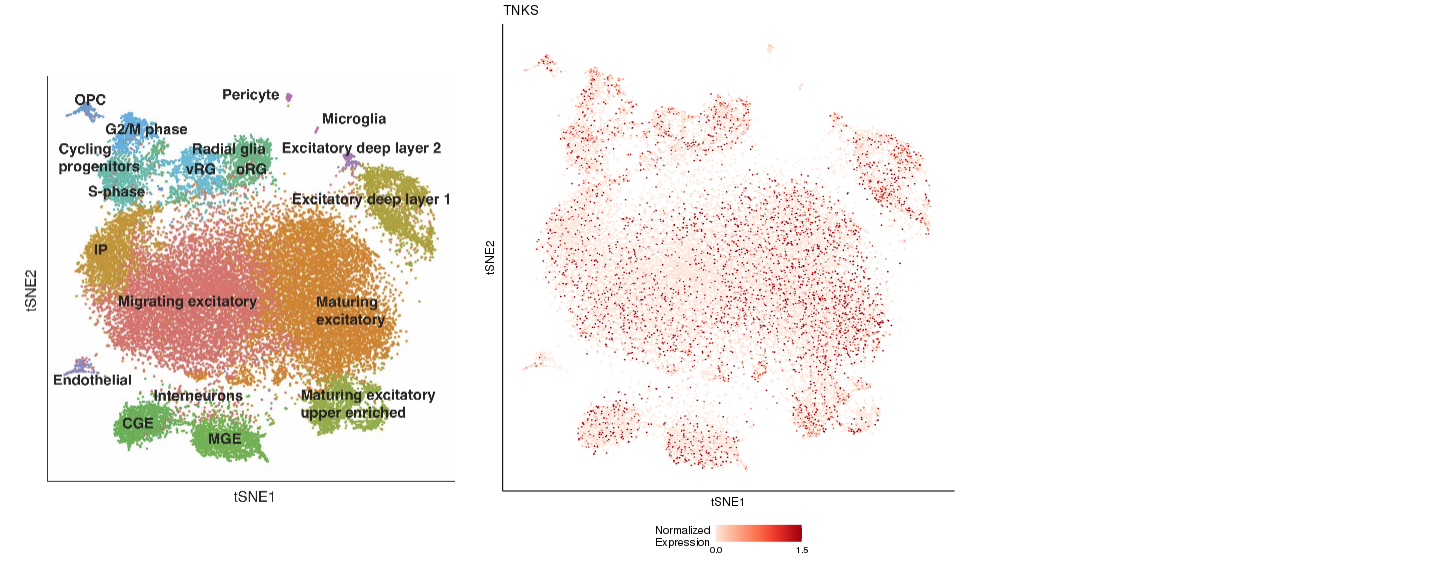
**Fig. S6. *TNKS* expression in different brain cell types.** Data was from the Cortical Development Expression Viewer (<http://solo.bmap.ucla.edu/shiny/webapp/>).

**Table S1. Genetic correlation between epilepsy and other neurological and psychiatric dieases**

| **Trait1** | **Trait2** | **PMID** | **Category** | **Ethnicity** | **Correlation** | **se** | **P** |
| --- | --- | --- | --- | --- | --- | --- | --- |
| epilepsy | Amyotrophic lateral sclerosis | 27455348 | neurological | European | 0.4346 | 0.108 | 5.74E-05 |
| epilepsy | Depressive symptoms | 27089181 | psychiatric | European | 0.1344 | 0.0502 | 0.0074 |
| epilepsy | Anorexia Nervosa | 24514567 | psychiatric | European | 0.1019 | 0.0409 | 0.0127 |
| epilepsy | Bipolar disorder | 21926972 | psychiatric | European | 0.202 | 0.0546 | 0.0002 |
| epilepsy | Autism spectrum disorder | 0 | psychiatric | European | -0.1621 | 0.0707 | 0.0218 |
| epilepsy | Schizophrenia | 25056061 | psychiatric | Mixed | 0.1408 | 0.0374 | 0.0002 |

**Data were from the LD Hub (**[**http://ldsc.broadinstitute.org/**](http://ldsc.broadinstitute.org/)**)**

| **Drug** | [**Interaction Type & Directionality**](https://www.dgidb.org/interaction_types) | **Sources** | **PMIDs (Pubmed IDs)** | [**Query Score**](https://www.dgidb.org/score) | [**Interaction Score**](https://www.dgidb.org/score) |
| --- | --- | --- | --- | --- | --- |
| [ETHOSUXIMIDE](https://www.dgidb.org/drugs/ETHOSUXIMIDE) | n/a | [**PharmGKB**](https://www.dgidb.org/sources/PharmGKB) | [**23859570**](http://www.ncbi.nlm.nih.gov/pubmed/23859570) [**21747585**](http://www.ncbi.nlm.nih.gov/pubmed/21747585)  [**28753467**](http://www.ncbi.nlm.nih.gov/pubmed/28753467) [**25155934**](http://www.ncbi.nlm.nih.gov/pubmed/25155934) | 4.38 | 1.03 |
| [LEVETIRACETAM](https://www.dgidb.org/drugs/LEVETIRACETAM) | n/a | [**PharmGKB**](https://www.dgidb.org/sources/PharmGKB) | [**23859570**](http://www.ncbi.nlm.nih.gov/pubmed/23859570) [**21747585**](http://www.ncbi.nlm.nih.gov/pubmed/21747585)  [**28753467**](http://www.ncbi.nlm.nih.gov/pubmed/28753467) [**25155934**](http://www.ncbi.nlm.nih.gov/pubmed/25155934) | 3.13 | 0.73 |
| [PHENACEMIDE](https://www.dgidb.org/drugs/PHENACEMIDE) | blocker (inhibitory), inhibitor (inhibitory) | [**TdgClinicalTrial**](https://www.dgidb.org/sources/TdgClinicalTrial) [**ChemblInteractions**](https://www.dgidb.org/sources/ChemblInteractions) [**TEND**](https://www.dgidb.org/sources/TEND) | [**3959032**](http://www.ncbi.nlm.nih.gov/pubmed/3959032) [**11752352**](http://www.ncbi.nlm.nih.gov/pubmed/11752352)  [**17139284**](http://www.ncbi.nlm.nih.gov/pubmed/17139284) [**17016423**](http://www.ncbi.nlm.nih.gov/pubmed/17016423) | 3.07 | 0.72 |
| [OXCARBAZEPINE](https://www.dgidb.org/drugs/OXCARBAZEPINE) | blocker (inhibitory), inhibitor (inhibitory) | [**ChemblInteractions**](https://www.dgidb.org/sources/ChemblInteractions) [**PharmGKB**](https://www.dgidb.org/sources/PharmGKB) | [**23859570**](http://www.ncbi.nlm.nih.gov/pubmed/23859570) [**21747585**](http://www.ncbi.nlm.nih.gov/pubmed/21747585) [**22591328**](http://www.ncbi.nlm.nih.gov/pubmed/22591328)  [**10845729**](http://www.ncbi.nlm.nih.gov/pubmed/10845729) [**28842369**](http://www.ncbi.nlm.nih.gov/pubmed/28842369) [**28753467**](http://www.ncbi.nlm.nih.gov/pubmed/28753467)  [**12737829**](http://www.ncbi.nlm.nih.gov/pubmed/12737829) [**25155934**](http://www.ncbi.nlm.nih.gov/pubmed/25155934) [**26555147**](http://www.ncbi.nlm.nih.gov/pubmed/26555147) | 2.41 | 0.57 |
| [ZONISAMIDE](https://www.dgidb.org/drugs/ZONISAMIDE) | blocker (inhibitory), inhibitor (inhibitory) | [**TdgClinicalTrial**](https://www.dgidb.org/sources/TdgClinicalTrial) [**ChemblInteractions**](https://www.dgidb.org/sources/ChemblInteractions) [**TEND**](https://www.dgidb.org/sources/TEND) | [**19557119**](http://www.ncbi.nlm.nih.gov/pubmed/19557119) [**20001433**](http://www.ncbi.nlm.nih.gov/pubmed/20001433) [**19948168**](http://www.ncbi.nlm.nih.gov/pubmed/19948168)  [**15511691**](http://www.ncbi.nlm.nih.gov/pubmed/15511691) [**11752352**](http://www.ncbi.nlm.nih.gov/pubmed/11752352) [**20025128**](http://www.ncbi.nlm.nih.gov/pubmed/20025128)  [**14704463**](http://www.ncbi.nlm.nih.gov/pubmed/14704463) [**18433351**](http://www.ncbi.nlm.nih.gov/pubmed/18433351) | 2.1 | 0.49 |
| [CARBAMAZEPINE](https://www.dgidb.org/drugs/CARBAMAZEPINE) | blocker (inhibitory), inhibitor (inhibitory) | [**ChemblInteractions**](https://www.dgidb.org/sources/ChemblInteractions) [**PharmGKB**](https://www.dgidb.org/sources/PharmGKB) | [**23859570**](http://www.ncbi.nlm.nih.gov/pubmed/23859570) [**21747585**](http://www.ncbi.nlm.nih.gov/pubmed/21747585) [**22591328**](http://www.ncbi.nlm.nih.gov/pubmed/22591328)  [**19289736**](http://www.ncbi.nlm.nih.gov/pubmed/19289736) [**20643904**](http://www.ncbi.nlm.nih.gov/pubmed/20643904) [**22292851**](http://www.ncbi.nlm.nih.gov/pubmed/22292851)  [**28753467**](http://www.ncbi.nlm.nih.gov/pubmed/28753467) [**22188362**](http://www.ncbi.nlm.nih.gov/pubmed/22188362) [**12391287**](http://www.ncbi.nlm.nih.gov/pubmed/12391287)  [**26314341**](http://www.ncbi.nlm.nih.gov/pubmed/26314341) [**15208697**](http://www.ncbi.nlm.nih.gov/pubmed/15208697) [**25155934**](http://www.ncbi.nlm.nih.gov/pubmed/25155934)  [**26555147**](http://www.ncbi.nlm.nih.gov/pubmed/26555147) [**20526191**](http://www.ncbi.nlm.nih.gov/pubmed/20526191) [**21561445**](http://www.ncbi.nlm.nih.gov/pubmed/21561445) | 1.59 | 0.37 |
| [TETRODOTOXIN](https://www.dgidb.org/drugs/TETRODOTOXIN) | blocker (inhibitory) | [**TdgClinicalTrial**](https://www.dgidb.org/sources/TdgClinicalTrial) [**GuideToPharmacology**](https://www.dgidb.org/sources/GuideToPharmacology) | [**17663442**](http://www.ncbi.nlm.nih.gov/pubmed/17663442) | 1.46 | 0.34 |
| [TETRACAINE](https://www.dgidb.org/drugs/TETRACAINE) | blocker (inhibitory) | [**ChemblInteractions**](https://www.dgidb.org/sources/ChemblInteractions) | [**23888118**](http://www.ncbi.nlm.nih.gov/pubmed/23888118) [**22053156**](http://www.ncbi.nlm.nih.gov/pubmed/22053156) | 1.2 | 0.28 |
| [LAMOTRIGINE](https://www.dgidb.org/drugs/LAMOTRIGINE) | blocker (inhibitory), inhibitor (inhibitory) | [**ChemblInteractions**](https://www.dgidb.org/sources/ChemblInteractions) [**PharmGKB**](https://www.dgidb.org/sources/PharmGKB) | [**23859570**](http://www.ncbi.nlm.nih.gov/pubmed/23859570) [**21747585**](http://www.ncbi.nlm.nih.gov/pubmed/21747585)  [**28753467**](http://www.ncbi.nlm.nih.gov/pubmed/28753467) [**16174788**](http://www.ncbi.nlm.nih.gov/pubmed/16174788)  [**25155934**](http://www.ncbi.nlm.nih.gov/pubmed/25155934) | 1.18 | 0.28 |
| [VERATRIDINE](https://www.dgidb.org/drugs/VERATRIDINE) | activator (activating) | [**GuideToPharmacology**](https://www.dgidb.org/sources/GuideToPharmacology) | *None found* | 1.1 | 0.26 |

**Table S2. The interactions between SCN1A and drugs**

**Data were from (DGIdb) (https://www.dgidb.org/)**

**Table S3. The interactions between SCN1A and drugs**

| **Drug** | [**Interaction Type & Directionality**](https://www.dgidb.org/interaction_types) | **Sources** | **PMIDs** | [**Query Score**](https://www.dgidb.org/score) | [**Interaction Score**](https://www.dgidb.org/score) |
| --- | --- | --- | --- | --- | --- |
| [DECOGLURANT](https://www.dgidb.org/drugs/DECOGLURANT) | modulator | [**ChemblInteractions**](https://www.dgidb.org/sources/ChemblInteractions) [**TTD**](https://www.dgidb.org/sources/TTD) | [**23976856**](http://www.ncbi.nlm.nih.gov/pubmed/23976856) | 6.58 | 11.96 |
| [LY404039](https://www.dgidb.org/drugs/LY404039) | agonist (activating) | [**ChemblInteractions**](https://www.dgidb.org/sources/ChemblInteractions) [**TTD**](https://www.dgidb.org/sources/TTD) | *None found* | 4.38 | 7.97 |
| [SPAGLUMIC ACID](https://www.dgidb.org/drugs/SPAGLUMIC%20ACID) | agonist (activating) | [**GuideToPharmacology**](https://www.dgidb.org/sources/GuideToPharmacology) | *None found* | 4.38 | 7.97 |
| [LY2969822](https://www.dgidb.org/drugs/LY2969822) | agonist (activating) | [**ChemblInteractions**](https://www.dgidb.org/sources/ChemblInteractions) | *None found* | 2.19 | 3.99 |
| [POMAGLUMETAD METHIONIL](https://www.dgidb.org/drugs/POMAGLUMETAD%20METHIONIL) | agonist (activating) | [**ChemblInteractions**](https://www.dgidb.org/sources/ChemblInteractions) | *None found* | 2.19 | 3.99 |
| [EGLUMEGAD](https://www.dgidb.org/drugs/EGLUMEGAD) | agonist (activating) | [**GuideToPharmacology**](https://www.dgidb.org/sources/GuideToPharmacology) | *None found* | 1.1 | 1.99 |
| [DIACETYLMORPHINE](https://www.dgidb.org/drugs/DIACETYLMORPHINE) | n/a | [**PharmGKB**](https://www.dgidb.org/sources/PharmGKB) | *None found* | 0.34 | 0.61 |
| [RISPERIDONE](https://www.dgidb.org/drugs/RISPERIDONE) | n/a | [**PharmGKB**](https://www.dgidb.org/sources/PharmGKB) | [**19451915**](http://www.ncbi.nlm.nih.gov/pubmed/19451915) | 0.14 | 0.25 |

**Data were from (DGIdb) (https://www.dgidb.org/)**

**Table S4. The interactions between SCN1A and drugs**

| **Drug** | [**Interaction Type & Directionality**](https://www.dgidb.org/interaction_types) | **Sources** | **PMIDs** | [**Query Score**](https://www.dgidb.org/score) | [**Interaction Score**](https://www.dgidb.org/score) |
| --- | --- | --- | --- | --- | --- |
| [PAMIPARIB](https://www.dgidb.org/drugs/PAMIPARIB) | n/a | [**TTD**](https://www.dgidb.org/sources/TTD) | *None found* | 1.46 | 10.63 |
| [2X-121](https://www.dgidb.org/drugs/2X-121) | n/a | [**TTD**](https://www.dgidb.org/sources/TTD) | *None found* | 0.73 | 5.32 |

**Data were from (DGIdb) (**[**https://www.dgidb.org/**](https://www.dgidb.org/)**)**
